# Copper Toxicity Links to Pathogenesis of Alzheimer’s Disease and Therapeutics Approaches

**DOI:** 10.3390/ijms21207660

**Published:** 2020-10-16

**Authors:** Hafza Wajeeha Ejaz, Wei Wang, Minglin Lang

**Affiliations:** 1 CAS Center for Excellence in Biotic Interactions, College of Life Science, University of Chinese Academy of Sciences, Yuquan Road 19, Beijing 100049, China; wajeehaejaz17@gmail.com; 2 School of Medical and Health Sciences, Edith Cowan University, Perth WA6027, Australia; wei.wang@ecu.edu.au; 3 College of Life Science, Agricultural University of Hebei, Baoding 071000, China

**Keywords:** Alzheimer’s disease, amyloid plaques, copper, oxidative damages, protein modification, neurodegeneration

## Abstract

Alzheimer’s disease (AD) is an irreversible, age-related progressive neurological disorder, and the most common type of dementia in aged people. Neuropathological lesions of AD are neurofibrillary tangles (NFTs), and senile plaques comprise the accumulated amyloid-beta (Aβ), loaded with metal ions including Cu, Fe, or Zn. Some reports have identified metal dyshomeostasis as a neurotoxic factor of AD, among which Cu ions seem to be a central cationic metal in the formation of plaque and soluble oligomers, and have an essential role in the AD pathology. Cu-Aβ complex catalyzes the generation of reactive oxygen species (ROS) and results in oxidative damage. Several studies have indicated that oxidative stress plays a crucial role in the pathogenesis of AD. The connection of copper levels in AD is still ambiguous, as some researches indicate a Cu deficiency, while others show its higher content in AD, and therefore there is a need to increase and decrease its levels in animal models, respectively, to study which one is the cause. For more than twenty years, many in vitro studies have been devoted to identifying metals’ roles in Aβ accumulation, oxidative damage, and neurotoxicity. Towards the end, a short review of the modern therapeutic approach in chelation therapy, with the main focus on Cu ions, is discussed. Despite the lack of strong proofs of clinical advantage so far, the conjecture that using a therapeutic metal chelator is an effective strategy for AD remains popular. However, some recent reports of genetic-regulating copper transporters in AD models have shed light on treating this refractory disease. This review aims to succinctly present a better understanding of Cu ions’ current status in several AD features, and some conflicting reports are present herein.

## 1. Introduction

Alzheimer’s disease (AD) is a multifactorial, complex brain disease defined by progressive cognitive decline, heterogeneity of behavioral presentations, and dementia in older people [1,2,3]. In 1907, Alois Alzheimer was the first to identify a mental decline with amyloid plaques and neurofibrillary tangles found in most dementia symptoms [4,5]. This disorder’s main risk factor is old age, because the elderly are more prone to diseases, affecting 10% of people aged 65, and this proportion rises by about three times for people aged 85 and older [6,7]. AD typically destroys neurons, and their connection with the brain regions such as the entorhinal cortex and hippocampus area, the parts of the brain essential in forming memories [8]. This disorder disrupts processes necessary for healthy neurons, such as communication, metabolism, and repair [9,10]. Ultimately, the disease is fatal. It is one of the leading causes of death [11] that we are currently unable to stop or cure because the underlying etiology is poorly understood at present [11,12].

Unfortunately, the treatment of AD has often been delayed in general because it is diagnosed only after prominent signs of cognitive deterioration [13], and this is all due to the lack of awareness of cognitive problems on the part of patients and patients’ families [14]. Clinical detection of this disorder is only possible when the symptoms are advanced enough to show visible behavior or cognitive changes [15]. There could be enough time to halt or slow this disorder’s development with early AD identification before complete onset [15]. Indeed, currently, there is no such treatment for AD [16], and approved drugs that have insignificant effects at altering the pathophysiological course of this disorder [17,18], due to the disease developing from a combination of lifestyle, environment, and genetic risk factors that affect the brain over time [19,20].

One of the most common neuropathological hallmarks of AD is the misfolding and aggregation of amyloid plaques-extracellular insoluble deposits of the β-amyloid peptides [21], and the intracellular formed NFTs (neurofibrillary tangles) [22], leading to the loss of communication between nerve cells, causes brain damage and shrinkage [23]. Posterior cingulated cortex (PCC) [24], entorhinal cortex (EC) [25], hippocampus (HIP) [26] (the first part to be affected by AD), middle temporal gyrus (MTG) (role in cognitive functions such as language processing), and superior frontal gyrus (SFG) (helps in memory) [27,28] are the regions affected in this multifactorial neurological disorder. Some studies have identified the impaired function of the middle temporal gyrus [29] and superior frontal gyrus in AD [30].

Extracellular deposits of Aβ peptides in Alzheimer’s are the main pathological events in AD [31,32,33,34]. Senile plaques or amyloid plaques mainly consist of small amyloid beta-peptides (Aβ) (up to 42 or 43 amino acids long) [35]. These are β amyloid precursor protein (APP) metabolites, derived by proteolytic sequential cleavage, first through β-secretase and then with γ-secretase, in the amyloidogenic pathway of producing peptides (Aβ), which contain 39 to 43 amino acids [36]. The APP (main isoforms, APP(695), APP(751), and APP(770)) is a type 1 transmembrane glycoprotein, which is essential for neurogenesis, neurite outgrowth, neuronal guidance, synapse formation, and repair [37,38,39]. The reason for neuritic plaques (senile plaques) forming in AD is due to irregularity between the production and removal of the beta-amyloid protein that accumulates [7]. Hence, the amyloid cascade hypothesis postulates that aggregation and accumulation of Aβ is the first pathological event in AD onset and initiates a cycle of adverse physiological changes that lead to neurodegeneration.

Another study has investigated Aβ aggregations in the senile plaques and co-localization of adenosine receptors in the AD [40]. Recently, some investigations have been done on adenosine, a purine ribonucleoside, because of its neuromodulator and neuroprotection function in neurological disorders [41,42]. It is present in all cells containing glia and neurons, initiates its biological process by four G-protein coupled receptors (GPCRs), namely, the A1, A2A,…A2BAR [43,44]. It has a role in regulating and integrating neuronal excitability, affecting many essential brain activities like sleep, memory, and neural plasticity [45,46,47]. Much research has analyzed adenosine effects via its receptors A1 and A2A in AD [48]. Nonselective blockage or modulation of these two receptors could protect cognitive impairment, making them innovative feasible therapeutic agents for AD [49]. Hippocampus, a brain region important for memory, learning, and neurogenesis [50,51,52], is one of the earliest affected brain regions that tends to exhibit the most rapid volume loss in the disease progression, and its pathology was found to be central to AD [50,53,54].

The hippocampus is a sensitive part of the brain to the dysfunctional homeostasis of transition metals, more so than any other brain region. Much research has also identified another brain part, the cortex, which is damaged by AD [55,56,57], linked with motor function, planning, organization, argumentation, feeling, and language processing [58]. NFTs are mainly composed of the microtubule-associated protein tau, predominantly expressed in the neurons under physiological conditions. This protein is mis-sorted into the somatodendritic compartment due to the tau sorting process’s failure, which is another essential factor that aggregates in AD [59]. Microtubules are essential components of a neuron’s cytoskeletal system, required for several fundamental cellular and dendritic processes, such as neuronal migration, polarity, axonal production, and differentiation [60,61]. Abnormal Aβ production might lead to the activation of tau mis-sorting, inducing tau pathology [62,63].

Multivalent metal ions such as copper (Cu) [64,65,66], zinc (Zn) [67,68], and iron (Fe) [69,70] are reported to be at higher levels in Alzheimer’s senile plaques [71,72]; while the connection of these metal ions with Aβ aggregation is still not well known. Indeed, some evidence from transgenic animal studies shows that Cu accumulates in senile plaques in the brains of 5 × FAD and Tg-SwDI/NOS2−/− mice models with neurodegeneration, as compared to PSAPP, where no Cu deposition has been seen among the mice with less neurodegeneration [73]. Much research has accumulated on Zn and Cu ions’ altered homeostasis as the central pathological hallmark [74,75,76] and shows the link of proteins related to Cu metabolism with this multifactorial AD [77].

Considerable research has suggested that Cu dyshomeostasis contributes to the onset of the most common neurodegenerative disorders besides AD, including Parkinson’s disease, prion-mediated encephalopathies, Huntington’s disease (HD), and amyotrophic lateral sclerosis (ALS) [78,79,80,81]. Hence, circumstances leading to a higher or lower copper concentration can be hazardous to health, such as Menkes diseases, a genetic disorder of Cu deficiency [82,83]. Furthermore, an autosomal recessive disorder, Wilson disease (WD), caused by defects of the ATP7B gene with excessive copper deposition in the body and patients’ brain examinations have shown copper concentration eight times greater than the controls [84].

Contradicting reports about the Cu concentration in AD has been reported. Some researches indicate a copper deficiency [85,86,87,88], while the majority show its higher level in AD, and therefore, reducing its level is required [89,90,91,92,93,94,95]. Investigations have grown exponentially in the neurodegenerative disorder fields over the past two decades. However, AD’s exact etiology is still not well understood, and as such, there is no successful therapeutic option available for this disorder to date [96,97].This literature review aims to present current knowledge regarding Cu’s role in AD. Towards the end, a short review of feasible therapeutics/strategies recommended for solving the problems associated with the metal’s implication in AD has also been discussed.

## 2. Copper Ion Implication in AD

Like other body parts, the brain contains many necessary transition metal ions, such as cobalt, copper, chromium, iron, zinc, and non-essential metals. Generally, the brain is the part of the body that contains the highest amount of transition metal ions content per weight. In comparison, the content of the copper ion in the brain is 0.004 g per kg [98]. It is an important chemical component of cell biology because it can receive and donate electrons. Once delivered and spread in a body, the cycle of Cu ions as the cupric ion (Cu^2+^) in its higher oxidation state and cuprous (reduced) form (Cu^+^), often joined to cuproenzymes with a small proportion as labile Cu, which was named as free or unbound Cu [99]. As a redox catalyst, Cu is necessary for many enzymes’ catalytic activity, regulating various cellular, biochemical, and regulatory processes. This metal plays an essential role in the catalytic centers of metalloproteins, electron transfer (ET) sites, and structural components.

Many studies have provided information concerning high serum levels of non-Cp–Cu, which results in reduced cognitive function, and the rate of mild cognitive impairment (MCI) to AD increased [90,100,101]. Postmortem biochemical analyses of the AD brain have revealed the reduced total soluble Cu levels, while its content within insoluble neuritic plaques is raised [102,103,104]. However, despite the decreased total Cu level in the central nervous system (CNS), elevated levels of redox-active exchangeable Cu are found in the Brodmann (BA46) and the temporal lobe (BA22) areas. AD cortical tissue has an increased propensity to bind exchangeable Cu^2+^ with increasing oxidative damage and neuropathological alterations, which have been seen in Alzheimer’s cases [105].

### 2.1. Copper and Amyloid-Beta Precursor Protein

Many authors have cited several models on copper implication in AD. Indeed, the most approved have put forward the “gain-of-function” of Aβ after binding Cu^2+^ ions [106]. Alternatively, current hypotheses suggesting “loss-of-function” of Aβ as the pathology of this disorder [94,107]. However, APP exports metal from neurons and a lower level of the soluble, functional Aβ monomer may lead to copper accumulation in the cell [107].

APP can bind to Cu^2+^ and reduce it to Cu^1+^ through its copper-binding domain (CuBD). APP can strongly bind Cu^2+^ to the N-terminal resulting in the decrement of copper ions [108]. Genetic studies of animal models have suggested that the APP-induced conversion of Cu^2+^ to Cu^+^ increases copper ion removal from the brain; this process could justify the point of why Alzheimer cases show lower brain level and higher Cu content in their serum-plasma [100,101,103,109,110]. However, Cu-binding with the N-terminal domain of APP may manage other functions of this protein, including synaptogenic function, stability, and metabolism [111,112,113,114]. Interestingly, the lower the copper content of the brain, the higher the ratio of endocytosed APP, and the generation of Aβ maybe works as a defense mechanism to stop the unnecessary loss of Cu [114,115]. Though newly produced intracellular Aβ can remove Cu, it probably leads to dyshomeostasis of copper ions and Aβ peptide deposition into plaques. This process results in neuritic plaques formation because Cu ions increase Aβ accumulation and cell damage due to the production of reactive oxygen species in AD [116,117,118].

Amyloid plaques or senile plaques mostly consist of the Aβ peptides, the essential peptide whose presence at a nanomolar concentration is shown by numerous studies in the cerebrospinal fluid (CSF) as well as in serum [119,120]. However, the TASTPM animal model study pointed out that the concentration of Cu in the Alzheimer’s brain does not link to plaques deposition [121]. The affinity of Cu^2+^ ions for Aβ peptides is very high [122,123], and it also increases the portion of beta-sheet and alpha-helix in Aβ proteins, which may be the cause of its aggregation [124]. So, β-amyloid deposition is the reason behind pathological alterations in AD, and its clearance when patients are immunized does not stop this disorder [125,126,127,128,129]. However, some scientific studies reported the presence of neuritic plaques in the brains of cognitively healthy elderly [130,131,132,133].

**Figure 1:**
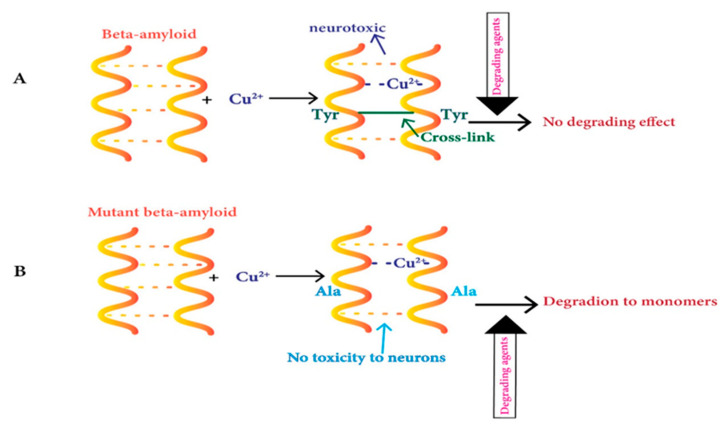
Copper’s role in the aggregation of Aβ peptides in the neuritic plaques of AD. (**A**) Cu^2+^ complexes with beta-amyloid peptides lead to dityrosine-linked β-amyloid dimer formation, which is neurotoxic and resists degradation into monomers. (**B**) Cu binding with the Y10A mutant peptide causes no neurotoxicity, dityrosine cross-linking, and degrades to monomers via degrading agents

The soluble oligomers obtained from the culturing of cells possess high chemical resistance and protect against its conversion into monomers via several degrading factors and maintain the presence of covalent cross-links in them [134,135]. Binding of Cu^2+^ ions increases dityrosine-linked β-amyloid dimers as observed in vitro studies of this neurological disorder [136,137,138,139]. This dimer structure switches from parallel to anti-parallel in the presence of Cu^2+^ and this process is regulated with the occupied binding sites of Cu [140]. Moreover, the same scholars later demonstrated that the nanomolar content of Cu^2+^ has no impact on peptide–peptide bonds of dityrosine-linked β-amyloid dimers [141]. Another study has shown that Cu^2+^ ions binding results in structural variations in the β-amyloid dimers, causing oligomer-defining interactions, including N-terminal interaction in them [142]. The mutant dimer does not make dityrosine cross-links because of tyrosine10 (Y10) mutation to alanine on Aβ, and it is not linked to neurotoxicity (Figure 1) [143].

A meta-analysis [144] and the following investigations [105,145] revealed a lower total Cu in AD, while the level of labile Cu is higher in most of the brain areas affected by this disease [105]. Alzheimer’s brain tissues and the cortexes of transgenic animals with severe brain damage showed the high Cu^2+^ ions-binding capacity [105,146]. Additionally, the APPsw/0 mouse model study reported parenchymal Aβ plaques, but no damage to neurons has been observed (Table 1) [147,148].

In relation to neuroinflammation, copper performs essential roles in the activation of microglia. However, there is insufficient data present about this. Scientific studies have suggested that Cu increases Aβ toxicity, and the microglia with fibrillar Aβ results in phenotypic activation, and the activated microglia is neurotoxic and causes neurodegeneration [160]. Cu-Aβ complex causes activation of microglia and the release of tumor necrosis factor-α (TNF-α) and nitric oxide (NO) in an NF-kappa B dependent pathway [89,161]. Recently, the study by Kitazawa (2016) [162] indicated that copper-Aβ complex attenuated microglial phagocytosis of BV2 and improved the release of TNF-α and interleukin-1 beta (IL-1β), which results in reducing expression of lipoprotein receptor-related protein-1 (LRP-1). Reduction in the level of LRP-1 leads to further impairment in the transcytotic Aβ clearance and increased neuroinflammation [163]. Indeed, a study showed that a trace level of Cu increases the Aβ induced neurotoxicity in the cholesterol-fed mouse through the inflammatory pathway; but, no effects of inflammation have been seen when treated with copper or cholesterol only [164]. Activated microglia expresses the ATP7A, also known as the Menkes protein (MNK), which is indicated to be gathered around the plaques by histological investigations.

Interestingly, the expression of ATP7A has been determined to be increased by interferon-gamma (IFN-γ), which is a pro-inflammatory cytokine but not by TNF-alpha or IL-1beta [165]. The inflammatory process linked with AD has been shown accompanied by the altered microglial copper homeostasis in the disease. Remarkably, the copper-deficient diet-fed mice showed symptoms of activated microglia and astrocytes, proposing Cu homeostasis is required under physiological conditions to stop neuroinflammation [166]. Moreover, some studies have suggested that copper homeostasis controls pro-inflammatory and anti-inflammatory phenotypes shift in microglia cell, by the nitric oxide regulation and disruption of S-nitrosothiol signaling [167,168]. However, further study is needed to understand the underlying etiology of how Cu controls the CNS immune responses, especially its function in the clearance of pathological hallmarks of AD, such as β-amyloid and tau, which may provide a new drug target for AD.

Cu^2+^ induced fibril formation at physiological pH because it is a highly pH-dependent process. However, amorphous aggregation occurs under the acidic environment [142,169]. Hence, the misfolding of Aβ40 and Aβ42 in the brain are neuropathological hallmarks of AD. Moreover, the molecular mechanism of its aggregation in vivo is still unclear. However, metal ions affect their deposition *in vitro*. Aβ42 aggregates much faster than the most common form Aβ40, and more toxic to neurons than Aβ40, even though Aβ42 differs from Aβ40 by only two (IA) amino acid residues at the C-terminal end. As Aβ40 contains more than one binding site of Cu, the second Cu^2+^-binding site interferes with the aggregation of Aβ40 to the amyloid fibrillar state in a proton-rich environment [152].

### 2.2. Copper and Tau Protein

**Figure 2:**
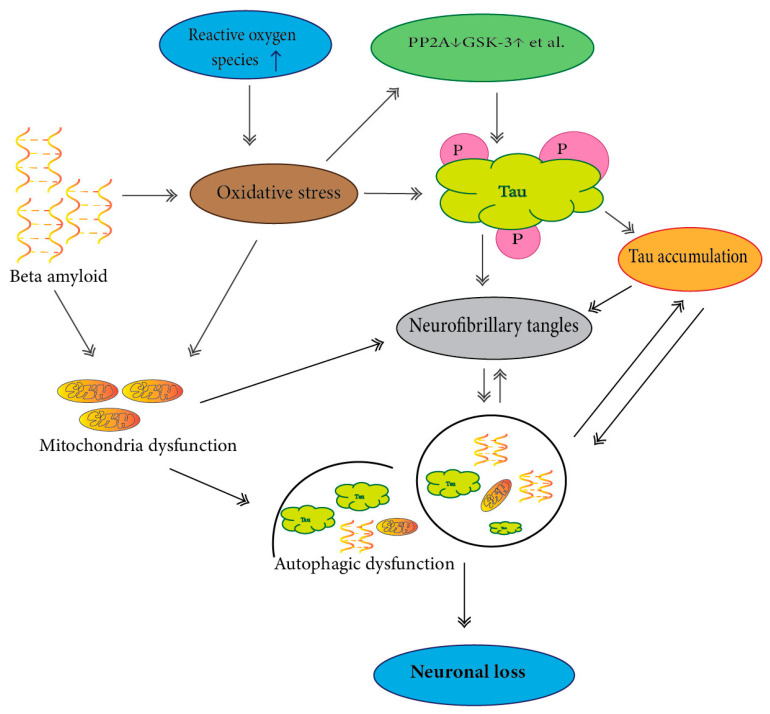
Dysfunction of autophagy and tau protein neurofibrillary tangles (NFTs) in the hippocampus of AD. Oligomeric Aβ-induced ROS production results in oxidative damage and mitochondrial dysfunction, in which hyperphosphorylated tau protein and NFTs produce through an imbalance of various protein kinases and phosphatases. These events lead to autophagic dysfunction and aggregated tau protein to neuronal loss in Alzheimer’s disease.

Autophagic-lysosomal flux is a lysosome-dependent cellular degradation program that plays an essential role in the clearance process of abnormally modified cellular proteins. Much data have suggested that endo-lysosomal/autophagic dysfunction is responsible for soluble oligomeric forms and insoluble forms of tau aggregation (Figure 2) [170,171].

The tau protein has also been investigated for its Cu binding, which plays an essential role in NFTs production [157,172,173]. Tau protein shows redox activity when it binds to copper, causing oxidative damage to the brain tissues [174]. However, despite tau as the main pathological hallmark of AD, only a small proportion of research has been done to check its link with Cu’s dyshomeostasis. Further studies are needed to evaluate Cu function in tau kinases and phosphatases and their role in cognitive impairment. These works will also increase our knowledge of the neuropathology of AD (Table 1).

### 2.3. Copper and ROS Production

Reactive oxygen species (ROS) generation is associated with a redox-active copper ion complex with aggregated Aβ, which has been identified to contribute to oxidative stress and damage to neuronal cells in AD [175].Copper-Aβ fibrils complex produces hydrogen peroxide (H_2_O_2_) in the presence of ascorbic acid, a biological reductant [175,176]. Increment in the ratio of (Cu-Aβ) leads to the production of H_2_O_2_, hydroxyl radicals (OH•), and misfolding of proteins (aberrant aggregates) shifts from amyloid fibrils to amorphous aggregates [116]. While initial studies have shown ROS being dangerous to causing neurodegeneration, the recently gathered data suggest some ROS action is necessary for cognition function and memory development [177,178,179,180,181]. According to some results, it has been suggested that the Cu-Aβ complex generates less ROS than unbound Cu ions [182]. Cu ions interaction with Aβ results in its accumulation under a slightly acidic environment and promotes ROS production in Alzheimer’s patients [183]. The production of ROS due to metal ions such as Cu leads to oxidative damages to Aβ peptide. This oxidized β-amyloid has been seen in senile or amyloid plaques during in vivo studies [184]. Some in vitro data imply that oligomeric and fibrillar forms of Aβ prevent hydrogen peroxide production at high concentrations of Cu^2+^. Additionally, amyloid fibrils produce less hydrogen peroxide than that in the oligomeric state [185].

However, the pro-oxidant function of the Cu-Aβ complex is not confirmed yet, because this complex is more effective in ROS generation than several tested biological relevant Cu-peptides and Cu-binding proteins [186] but less efficient than loosely-bound Cu [182,187,188,189]. It is usually stated that hydrogen peroxide production is a two-electron oxidation process; however, current research has indicated the production of superoxide (O^2−^) as an intermediate in hydrogen peroxide formation via Cu-Aβ complex and oxygen [190]. Cu is redox-active and, when bound to Aβ, catalytically cycles between the Cu^+1^ and Cu^+2^ oxidative states to generate ROS such as O•^−2^, OH•, and H_2_O_2_. Thus, the coordination of amyloid-β with Cu ions plays a significant role since ROS generation is a metal-catalyzed process, termed as a catalytic in-between state [191]. Therefore, computational studies have also examined Cu’s role in that state and its reactivity to the substrates such as oxygen or hydrogen peroxide [192,193,194].

Toxicity due to Cu ions in AD has been linked with the oxidant form of Cu ions such as Cu^2+^ [65,195]. Considerable studies found that the elimination of Cu^+^ from Aβ inhibits the production of Aβ oligomers and oxidative damage [196], and Cu^1+^ has a stronger affinity to monomeric Aβ peptide than Cu^2+^, which leads us to propose that Cu^1+^ cation is principal in the oxidation state in vivo [197].

Contrarily, the same metal ions also exist as catalytic metal ions, like Cu in SOD1, where they stop producing the H_2_O_2_. This also proves the value of coordination compounds. Copper can be in both pro-oxidants and antioxidants, which depends on its coordination position in compounds. However, in AD, higher production of ROS or less activity of the enzymes which degrade ROS results in an imbalance of pro-oxidants and antioxidants form, which cause oxidative damage on biomolecules [176]. Therefore, it is now clear how important it is to regulate copper ions’ metabolism in terms of content, transportation, storage, and association with active sites.

Abnormal Cu homeostasis increases the levels of free or loosely bound copper, which often produces ROS [98]. These ions can also attach to off-target biomolecules and disrupt their functional roles, leading to higher chances of oxidative damage.

**Figure 3:**
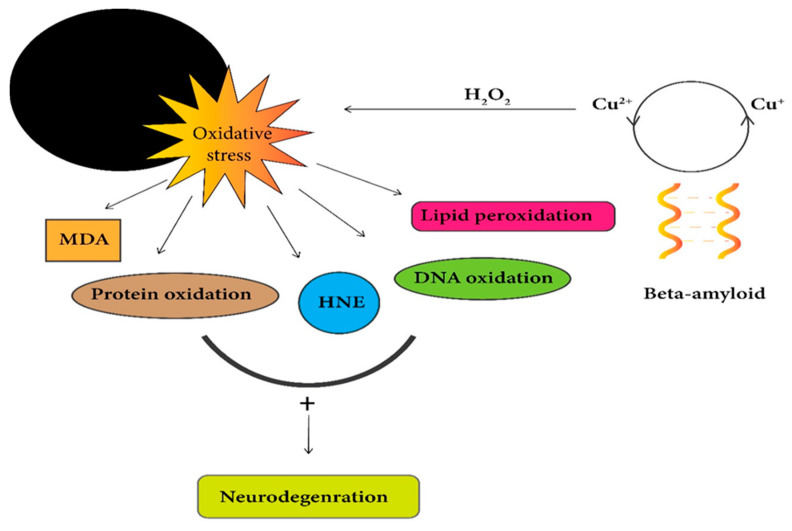
Redox cycling of Cu^2+^/Cu^+^ with Aβ peptides leads to the production of hydrogen peroxide. Unstable reactive oxygen species (ROS) production from H_2_O_2_ results in oxidative stress, leading to mitochondrial dysfunction, oxidative cellular damage, and neuronal loss. Cytotoxic end-products of lipid peroxidation malondialdehyde (MDA) and 4-hydroxy-2-nonenal (HNE) promote cell death.

Perhaps the Cu-Aβ complex is directly linked with ROS generation, so most of the studies have been associated with Cu-Aβ complex, suggesting a direct link between AD and oxidative damage [182]. Remarkably, all of the above data highlights the point that Cu is highly toxic in excess amounts and responsible for its participation in a redox-cycling reaction, which produces ROS that results in much damage to biomolecules such as carbohydrates, nucleic acids, lipids, and proteins. So, a higher level of free Cu ions causes more toxicity to the cells and eventually leads to cell death. Therefore, cellular Cu should be tightly controlled (Figure 3) [99].

### 2.4. Copper Deficiency and Cholesterol Rich in AD

A lower level of net copper was observed in the TgCRND8 mice model, with parenchymal amyloid aggregation but no loss of neurons [148,198]. The reason for early-onset familial AD is mutations in genes of proteins essential for mammalian systems in copper ion uptake [199]. As discussed before, a meta-analysis indicates a copper deficiency in the brain of Alzheimer’s cases [144]. Another study also showed a copper deficiency in the deceased Alzheimer’s brain’s defective regions with dementia symptoms [18]. Cu concentration of the elderly has a direct relation with Aβ aggregation [86]. While proteolytic cleavage of APP is a two-step pathway; non-amyloidogenic APP processing pathway and amyloidogenic pathway, in the presence and deficiency of Cu, respectively. Some results suggest the interaction of copper ions with a γ-secretase complex can inhibit amyloid production [200].

Based on the results of comparing blood copper levels of AD patients with healthy controls, which shows a significant reduction in copper ion, it has been hypothesized that Cu deficiency can lead to pathological hallmarks of AD [201,202]. An alternative study, which describes meta-analyses results of the copper quantification in serum-plasma and the brain, suggested Cu deficiency in the brain is a symptom of Cu dyshomeostasis, which relates to Wilson’s disease [203]. While, the dietary copper addition leads to an increase of intracellular copper concentration in APP/PS1 AD mice [155], which has been shown in parenchymal Aβ plaques, a decrease of AD pathology, but no loss of neurons seen [147,148]. Interestingly, some results showed that in AD patients, Cu deficiency does not have any link to their diet [201].

There is substantial proof about the amyloidogenic pathway’s connection with lipid raft, which is a particular cholesterol-rich microdomain. Although the deposition of Cu ions is associated with their cellular deficiency, the Cu level in lipid rafts has been inversely associated with the cellular Cu level, the simultaneous enrichment of Aβ and Cu within lipid rafts leads to higher redox-active Cu-Aβ complex formation in the absence of Cu conditions of AD [114,204]. Seemingly, a high-cholesterol rich diet plays an essential role in the AD pathology. Indeed, many investigations determined that lipids are a necessary part of this disorder [205,206,207]. Higher Cu^2+^ and lipid content in the neurodegenerative diseases have also been described by some recent scientific studies [208,209]. When the transgenic AD mice treated with Cu and cholesterol-fed diet, the ratio of Aβ42/Aβ40 increased, and a significant difference in the visuospatial memory was identified [208]; furthermore, in the rabbit brain, Aβ accumulation increases with the feeding of cholesterol food and Cu containing water (Cu ion in the form of Cu sulfate) [210].

**Figure 4:**
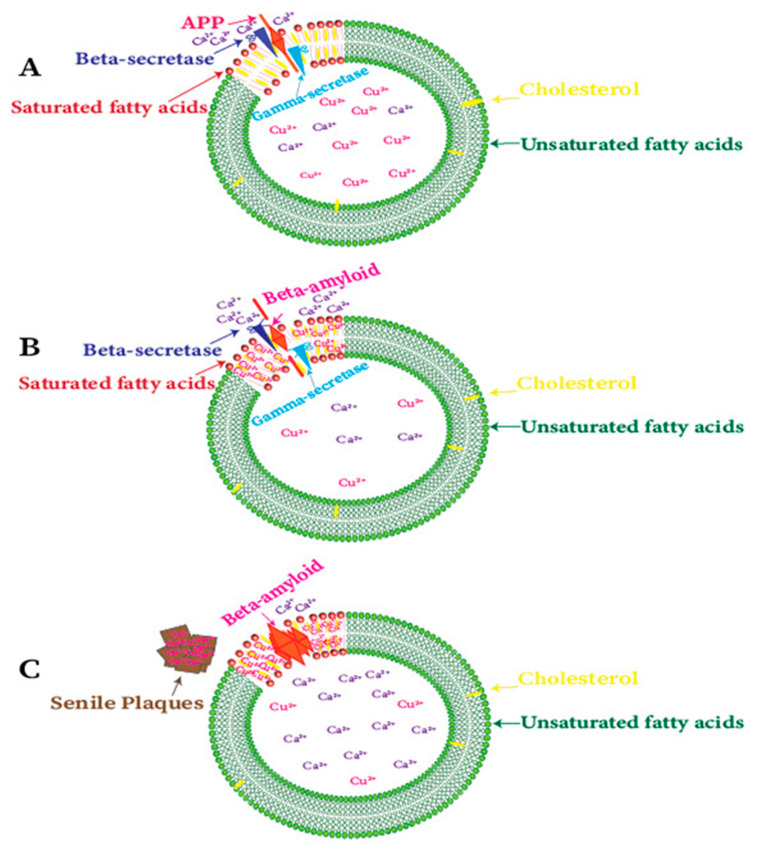
Schematic representation of the effects and correlation between Cu and cholesterol-rich lipid rafts in Alzheimer’s disease. (**A**) The enzymes that are present in lipid rafts are responsible for the cleavage of APP to Aβ peptide. (**B**) Cu deficient AD brains lead to copper accumulation in lipid rafts, and rising concentrations of Cu results in higher Aβ production due to an increase in β-secretase activity. (**C**) Calcium-permeable pores formed by small oligomers of Aβ peptides. These pores are calcium channels and disrupt cellular Ca^2+^ homeostasis, eventually leading to neuronal death.

Cholesterol-rich regions have also been detected for the enzyme activity of the cleavage of APP to amyloid proteins in AD brains [114,211]. Amyloid proteins attached to the plasma membrane surface and the Ca^2+^ ions help to penetrate the phospholipid bilayer [212]. The formation of Aβ22-35 channels is a cholesterol-dependent process and regulated with small cholesterol (~30 mol%) in phospholipid membranes. However, these channels cause an imbalance in Ca^2+^ homeostasis in neuronal cells and result in the bring-up of the Ca hypothesis of Alzheimer’s disease (Figure 4) [213,214]. Contrarily, Cu ions do not cause neurotoxicity in the absence of amyloid peptides [153]. Earlier unsuccessful therapeutic efforts and recent results about the aggregation of Aβ peptides in the cholesterol-rich regions (lipid rafts) lead to a different hypothesis that soluble Aβ oligomers (AβOs) associated with the cell membrane are responsible for neurotoxicity in AD [215,216,217].

## 3. Contradictory Results about Copper Level in AD

In the body cells, Cu is absorbed through a high-affinity copper transporter Ctr1, incorporating cuprous (Cu^+^) ions from the intestinal microvilli’s surface. Little is known about Cu^2+^ absorption, which is probably absorbed by divalent metal transporter 1 (DMT1) or other shared metal transporters [218]. Ctr1is responsible for the majority (~70%) of Cu import into mammalian cells, from which Cu is passed to glutathione, which carries Cu through the cytoplasm [219]. The absorbed copper ions will be targeted to Cu-binding chaperones and enzymes in different cell compartments such as cytosolic, mitochondrial, and Golgi. In the cytosol, Cu chaperone for superoxide dismutase 1 (SOD1), CCS, mediates Cu^+^ loading. A recent study suggested that the direct transfer of copper from Ctr1 to chaperones and then passing it to SOD1 is via forming a Ctr1-CCS-SOD1 complex [218]. Besides CCS, soluble copper chaperones such as Atox1 and Cox17 can also escort Cu^+^ from Ctr1 in the cytosolic pool to facilitate copper supply to their specific target compartments [220].

Consequently, in the absence of Ctr1, other pathways to absorbed Cu ions are unavailable to the organism because of the sequestration of copper in the sub-apical vesicles. This has been confirmed by making the intestinal epithelial cell-specific knockout of the Ctr1 (Ctr1int/int) mice, which manifested severe Cu deficiency, and the majority died within three weeks of post-birth [221]. A considerable portion of ingested cuprous ions are passed into circulation in enterocytes to reach different tissues by Atox1/ATPase routes. The mouse model with inactivated ATOX1/ATP7A routes showed defects in Cu distribution, which leads to pathological variations in many organs, especially the brain [222].

In the CNS, Cu deficiency has been found in the hippocampus and amygdala regions of Alzheimer’s patients, which causes severe histopathologic alterations in AD. Additionally, scientific research has put forward that the frontal cortex tissue of Alzheimer’s patients had an increased susceptibility for exchangeable copper (CuEXC), which is associated with the overproduction of free radicals (ROS) in AD [223].

In the CSF of the AD patients, there is no significant change in Cu concentration as compared to that of the healthy cases (HC) [224]. Furthermore, within peripheral fluids, abnormal homeostasis of copper ions has been intensively investigated. The relevant data point to increased [224,225], decreased [88,226], or unchanged [227] serum or plasma Cu in Alzheimer’s patients. Many other scientific analyses have also reported excessive free or diffusible copper in serum [224,226,228]. However, Rembach (2013) has suggested the possibility of decreased non-CP copper levels-copper that is not bound to ceruloplasmin in mild cognitive impairment (MCI) and AD, which leads to a decline of copper-dependent biochemical activities in AD [229], such as reducing SOD1 activity of erythrocytes [88].

Cu association for AD is ambiguous as some substantial researches showed Cu deficiency in AD and, hence, it is required to increase Cu levels [86,87,88]. In contrast, many different scientific pieces of evidence demonstrated Cu overload, and thus it is necessary to reduce it [90,91,92,93,94,95]. The main updated explanation so far is that the abnormal Cu homeostasis is due to an increment in the labile Cu ions and a reduced attachment to proteins [107,174].

Until 2012, the published contradictory scientific researches fueled the debate of copper concentrations in AD. So far, to check Cu levels in various biological specimens of AD patients, such as serum, plasma, and CSF, six meta-analyses have been done during the past six years. Studies published from 1984 to 2017 have been included in these meta-analyses [100,101,224,230,231,232], which give unambiguous results: overall and unbound Cu both are present in higher concentrations in the serum-plasma samples of the AD patients compared to that in the healthy cases [230]. According to the very recent meta-analysis, which includes a total of 35 pieces of research: eighteen report an increase, fourteen show no change, and one reports a decrease in Cu level in the serum-plasma of this disorder [232]. Subsequently, three more studies have been published, stating increased Cu^2+^ ions level in Alzheimer’s compared to that in the healthy controls [233,234,235].

These recent researches have contributed considerably to the explanation of the previous controversy. In blood, a higher level of free plasma Cu, which has been identified in 50–60% of Alzheimer’s patients, can explain the higher level of serum Cu in AD [145,174,233,236]. Another earlier research also observed an increased concentration of serum copper ions in a special kind of AD (Alzheimer’s disease epsilon four apolipoprotein E allele carriers) [237]. According to some scientific investigation, a genetic basis may be the reason for this particular type of AD [237,238,239,240].

## 4. Therapeutics to Tackle Copper Ions in AD

Despite the exponential growth of scientific literature published in the neurodegenerative disorders area, especially for AD, the exact etiology of AD is still not well understood. To date, there is no successful therapeutic option available for this disorder [96,241]. While there is no cure, there are five FDA-approved medications to cope with the symptoms of AD, which may prevent this disease from getting worse over time [242].

*In vitro*, removal of Cu^2+^ from Aβ prevents its accumulation [243,244,245], leads to its degradation, stops hydroxyl radical (•OH) production and oxidative damage, and finally reduces cell death [245]. For the effects as mentioned above, researches have suggested potential metal chelation therapy for AD [246,247,248,249,250,251,252]. Nevertheless, the challenge is to build selective and specific metal chelators, as metal ions play crucial roles in Alzheimer’s brains. The first metal chelator made for arsenic toxicity in the 1940s was 2,3-Dimercaptopropanol (BAL) [253,254]. Much later, followed by the same approach, the first-generation of metal chelator, a lipophilic small molecule clioquinol (5-chloro-7-iodo-8HQ or CQ) was introduced at the end of the 1990s [244,255]. Transgenic mouse models treated with CQ showed promise by reducing Aβ accumulation by 50%. CQ reduced Aβ aggregation during Phase II trials and improved cognitive behavior, but failed to provide sufficient evidence of a positive clinical benefit in a larger clinical trial [256,257]. Furthermore, patients exhibited some severe side effects, including neurotoxicity and mutagenicity; therefore, further clinical trials of CQ were stopped.

The most progressive chelator so far is PBT2 (5,7-dichloro-2-((dimethylamino)methyl)) [258], a second-generation of scaffold-based chelator, which has been inspired by CQ and also showed excellent antioxidant properties [259,260,261]. It is a more effective Zn/Cu ionophore than CQ, which could decrease H_2_O_2_ formation, have greater BBB (blood-brain barrier) permeability, higher solubility, and could also inhibit Cu and Zn induced Aβ accumulation in vitro [261,262]. PBT2 treatment targets metal-induced damage [263], and most importantly, it prevents the loss of necessary metal ions from the body such as the kidney, liver, lungs, and brain [241,264]. It also shifts the Alzheimer’s phenotype within days by reducing insoluble Aβ levels by ~30% [261] and alters tau and synaptophysin protein levels’ phosphorylation. Interestingly, a lowered level of insoluble total and elevated levels of the soluble total tau has been shown in the treatment with PBT2 [259]. Despite the effects mentioned above, the results of human clinical trials are not up to the mark according to some studies [241,265]. However, some scholars have denied this idea [266]. Results from the phase IIb, the randomized clinical trial, were not as promising even though phase Ib/IIa preclinical trials demonstrated significant reductions in Aβ levels and improvement in various aspects of cognitive functioning. 

The research in the Tg 2576 transgenic mice model has shown parenchymal plaque [147,148], indicating that metal chelators help slow disease progression and remove Cu ions only helpful in the initial stages of the AD [267]. While PS1 and PS2 play roles in Cu^2+^ uptake, tissue-specific knocking down of the single presenilins ortholog (*PSN*) in *Drosophila* reduces Cu^2+^ levels and increases its susceptibility to oxidative insult [199]. It was observed that the silencing of *PSN* in flies had less sensitivity to excess dietary Cu due to the reduced copper uptake. BLOC-1 physically interacts with ATP7A, and disruption of the *Drosophila*’s dysbindin/BLOC-1 complex affects copper homeostasis in both mammalian cells and *Drosophila* [268].

Different approaches have been used to treat the pathological hallmarks of the multifactorial AD due to Cu dyshomeostasis, including the metal chelation therapy [261,269,270,271]. Restoring the intracellular copper decreases β-amyloid production, which was found through a mechanism that depends on the activation of phosphatidylinositol 3-kinase (PI3K)/PI3K-Akt pathway, and JNK (Jun N-terminal kinase) [200,272]. Moreover, lately, studies have observed increased intracellular Cu inhibited AD-causing Aβ peptide by direct targeting of presenilin (PS1 or PS2) subunits and nicastrin (NCT) in the γ-secretase complex [64,200,273]. Hence, higher intracellular Cu levels can improve cognitive function as well, by preventing β-amyloid aggregation and tau phosphorylation [155,274,275]. There is proof of the bis(thiosemicarbazone) copper(II) complex having the immunomodulatory potential [276,277], and greater BBB permeability. It inhibits microglial as well as astrocytic inflammatory responses and also has a role in the decrement of bacterial lipopolysaccharide (LPS) induced inflammation [278]. Some researchers have suggested that excess dietary Cu intake increases AD risks, and diets with measured copper should be supported. Additionally, a study conduct with a small amount of Cu in drinking water results in rising levels of amyloid peptides in the brain [162,207], a process that appears to be linked with dysfunction of LRP1-mediated efflux of Aβ from the brain [279] in vascular smooth muscle cells [280,281]. The median intake of copper from food among children and adolescents aged 2–19 years, the recommended daily allowance (RDA) ranges from 800 to 1000 mcg/day. In adults aged 20 and older, 1400–1700 mcg/day is recommended. Despite the difficulties, balancing Cu homeostasis has numerous advantages, and can be a potential drug target for this progressive, neurological disorder [89,156,282,283,284].

For other neurological disorders, such as Wilson’s disease (WD), a different chelating agent, tetrathiomolybdate (TTM, an ammonium salt), appears to be a promising alternative, which can act by inhibiting copper uptake. TTM had the advantages of being fast-acting and did not lead to neurological deterioration in WD patients. It can restore normal copper balance without increasing serum “free copper” within several weeks compared to other copper chelators or zinc salts requiring several months [285]. However, the ammonium formulation has been proven too unstable for routine use, so clinical experience with them remains limited. Of note, bis-choline salt of TTM, WTX101, has recently become available on a named patient basis in the USA and Europe. This complex is more stable than TTM and phase III FOCUS study compared to standard of care (SoC) in WD patients was started in 2018, with results expected in 2020 [286].

## 5. Multifunctional Chelators (MFCs) to Control Metal Mediated Abnormalities

**Figure 5:**
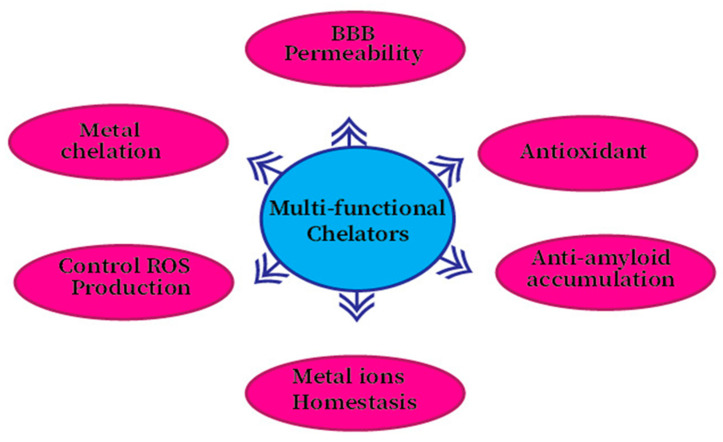
Multifunctional compounds (MFCs) target for Alzheimer’s Disease.

Because multiple pathological variables are involved in the pathology of AD, therapeutics or drugs target a single mechanism that is not enough to treat this disorder. New therapeutics or medicines that can target multiple factors at the same time can be beneficial for patients suffering from neurodegenerative disorders. It is now undeniable that the next generation of therapies should have the ability to target the different causes of disease progression at the same time [287]. Neurodegenerative disorder drugs must have more than one of the following properties to be useful for these diseases such as control of the production of ROS, the ability of metal chelating, and greater BBB permeability, a decrease of β-Amyloid peptide deposition (Figure 5), and last but not least, of regulating enzymes associated with the mechanism of the disease, for example, acetylcholinesterase [288,289].

**Figure 6:**
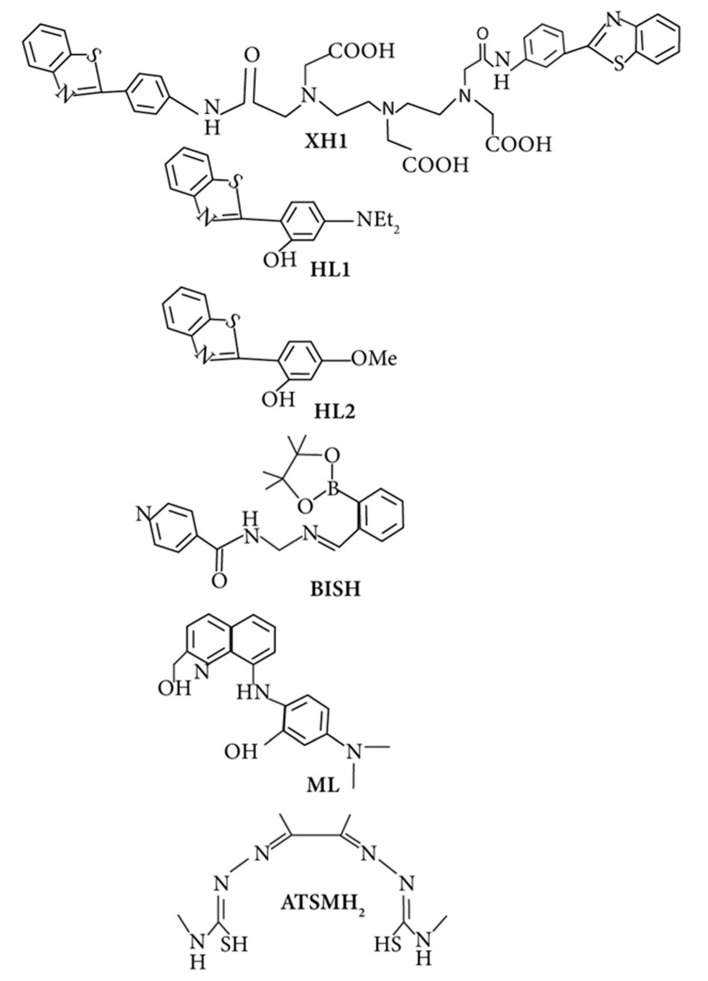
Structure of bifunctional chelating agents from various research groups’ reports as discussed in the text.

Bifunctional metal chelators (BFCs) were suggested to treat multifactorial AD because they have the ability of both metals chelating and binding with amyloids. Substantial research has been made in this field in the last decade [259,263,290,291]. The fluorescent dye thioflavin T (ThT), also known as Basic Yellow 1 or CI 49005, has been widely used to detect amyloid fibrils [292] in both in vivo and in vitro studies. The first bifunctional chelator designed was XH1 (Figure 6), connecting various molecular fragments of different specificities to make a hybrid molecule [293]. Its structure is composed of two-terminal thioflavin-T-derived moieties, which are attached by a DTPA (diethylene triamine penta-acetic acid) binding unit.

Besides metal ions’ chelation role in Alzheimer’s, new chelating molecules such as phenyl benzotriazole followed by the same design principle of thioflavin-T(ThT), correlating them with dipicolylamine or pyrinophane type metal chelators have been reported by the studies [263,287,291,294]. Several studies have analyzed the deposition of Aβ1-42 in the deficiencies and presence of essential metal ions [291,295]. Franz and co-workers have used persuasive strategies to design prochelators in 2006. These chelators only work in the presence of oxidative stress and inhibit essential ion loss like Cu and Zn of metalloproteins. Followed by the same approach, boronic ester (BSIH), an excellent first-generation prochelator metal affinity group, was composed. It works as an iron chelation with salicylaldehyde isonicotinoyl hydrazone (SIH), in the presence of H_2_O_2_ [296].

Various analogs of boronic ester, such as boronic esters (BSIH, BSBH) and acids (BASIH), have been investigated to check their performance as metal chelators [297]. Currently, many promising molecular scaffolds using the same strategy are exploring their effect on the multifactorial AD [298,299,300,301]. Choi (2011) and Hindo (2009) [302,303] reported the derived compounds and analyzed their impact on metal-binding properties, β-amyloid deposition both in deficiency and the presence of metal ions. Currently, small novel compounds such as 2,2-bipyridine (bpy) derivatives (1–4) and other N,N-dimethylaniline including novel N-bidentate ligands, have been described to show good results for the treatment of multifactorial AD [304,305]. The effects of several flavonoids such as myricetin and EGCG (epigallocatechin gallate) have also been tested for this disorder [306,307,308].

While Orvig described salen-type Schiff-bases in addition to other chelating agents for the first time, it has also been connected with carbohydrate moieties [259,309,310,311,312]. In vivo investigation of the diacetylbis(N(4)-methylthiosemicarbazonato) copper(II) (CuII(ATSM)) (Figure 6) compound has seen its protection against nitrosative damage of peroxynitrite and an increase of survival in the ALS mouse models [313]. Unfortunately, it was not studied much for metal chelation and protection against oxidative damage in AD. Many multifunctional molecules have been rationally designed to simultaneously resist and target various pathological hallmarks of the brain disorders, and their effects have been checked and reviewed by many studies [290,314]. Finally, despite all tremendous efforts that have been made for the success of multifunctional molecules as a potential therapy, only a handful of them show promising results in human clinical trials. Indeed the usage of these compounds to target the multi-mechanisms of the neurodegenerative diseases, its potential causes of failure also need to be understood, which has been reviewed elsewhere [122,265]. Overall, it is an effective strategy and will hopefully provide a better therapeutic option for Alzheimer’s cases when the compounds made become more tissue targeting specific.

## 6. Efficacy of Therapeutic Chelation

There are a large number of metal chelators that have been developed to cure AD. Indeed, only a few of them made their way to clinical trials [315,316]. To discriminate the bulk of chelation therapies, which mostly link with the release of heavy metal poisoning, these used therapeutic chelators have been named ionophores, metallochaperones, and MPACs (metal-protein attenuating compounds). CQ (PBT1) and PBT2 are the most popular MPACs for AD, both were designed based on old chemistry with different applications, and the term MPACs was popularly used because it was a belief that PBT1 and PBT2 cause deaccumulation of β-amyloid plaques loaded with Cu and Zn ions [317,318]. Terdendate ligands (L), such as PBT2, make bonds in a 1:1 ratio (Cullin 1), and a distortion occurs at 5-coordinate 1:2 ratio (Cullin 2) form, while the copper(II)-bound form of this class terdentate 8HQ, comprising peptides and side chains of proteins is predicting a ternary metal ion complex.

The word “ionophore” was used for a large number of cellular metal uptake experiments in vitro [261,319,320], while the name “metallochaperone” has now been suggested most of the times. Ionophores constitute a distinct subset of metal-binding drugs capable of moving multiple members of a given ion across cell membranes. The main difference lies between chelator and ionophores is in the functional result of the metal complex. Traditional medicinal chelating agents result in excretion of the absorbed metal from its receptor site into the system where it cannot exert toxicity and can make them bio-unavailable. In contrast, ionophores generally form lipophilic metal complexes that make the membrane permeable to specific ions, creating a more or less selective channel to particular ions. Hence, there is a possibility that 8HQs as carrier ionophores can work in the hydrophobic environment of several plasma membranes (PMs), and the Cu, which is not removed from 8HQs ligands, causes localization to phospholipid bilayer, and this results in 8HQs interference with bonds of heavy metals of essential regulatory enzymes [321,322,323] due to the formation of the ternary complex. Interestingly, the generation of ROS can be detected by adding such ligands to the culture of neural stem cells [324], in contrast to the founding principle of therapeutic chelation therapy [325].

Some therapeutic benefits of 8HQ therapeutic chelators have been suggested by transgenic animal studies of AD [261,319]. Indeed, chelation therapy in human clinical tests has not provided any satisfactory results so far. Chelation therapy using D-penicillamine has also not presented any proof of improving disease pathology and has to stop earlier in initial phases due to its side effects, causing some to question the usage of 8HQs [326]. Independent evaluation of the human clinical trials from 2006 to 2014 using 8HQs, frequently reported no advantage to AD patients [327,328,329]. Despite all these discouraging signs, the hypothesis is that 8HQs was successful in two human clinical trials. Post-hoc study of the Phase 2A trials claimed that PBT2 improves cognition in AD [330], though, the results of this clinical trial are in question. Another study by Ayton (2013) [331] also claimed the positive outcome of clinical trials. Therefore, some researchers were still showing promise for these compounds [325,332].

In short, according to Drew (2017) [265], there is a preference in describing results of clinical trials of Cu chelation as positive and helpful for Alzheimer’s cases, which results in continued checking of different chelators for the well-defined targets to treat the dyshomeostasis of metals in AD.

## 7. Conclusions

Collectively, studies strongly advocate that dyshomeostasis of Cu, leads to the onset and progression of AD. Earlier researches have recognized amyloid plaques as toxic factors in AD. Though 20–40% of healthy cases have amyloid plaques, as illustrated by some studies [333]. Furthermore, cell death often leads to amyloid plaque formation in the brain. While mounting evidence implicates ROS in the AD etiology, loosely bound copper ions are very efficient catalysts for ROS generation by a copper-amyloid complex [105,334].

Some studies indicate an increased liable pool of Cu in the brain [105,230] responsible for Cu deficiency. The reason of Cu deficiency seems to be an essential factor in AD. Copper deficiency leads to Cu enrichment in lipid rafts, so maybe an elevation in lipid raft domains could be the reason for Cu deficiency in the brain; thus, lipid raft domains could be an efficient drug target. Studies indicated that the disrupted lipid rafts (by omega-3 fatty acids) slowed the progression of AD [215,335]. Another direction for research depends on the feasibility of developing novel therapeutic approaches to work against this disease.

The proposal for direct chelation therapy of Cu ions to work in this disorder is still in discussion [265]. Support for the lowering cellular Cu levels comes from the *Drosophila* model of AD, where although copper chelation or genetic knockdown of copper transporters (Ctr1C) decreased the expression of Aβ degrading proteases but rescued the toxic phenotype [336]. Similar results were also observed by silencing the expression of Ctr1B, or when copper exporter DmATP7 [336] and dMTF-1 or MtnA [94] were overexpressed in the nervous system of the Aβ transgenic flies. These flies exhibited improved neurodegeneration, locomotion, longevity, and a reduction in Cu-Aβ complex-induced oxidative stress.

Furthermore, in parallel, antibody-based treatment for Aβ aggregation is now developing and providing safe results as well [337]. Research organizations should come to the same standpoint regarding the experimental requirements and procedures to be used, to avoid different and ambiguous results for such serious matters. In this context, all the struggles for a better understanding of AD pathology’s molecular mechanisms and developing innovative therapeutic approaches should be appreciated.

## Figures and Tables

**Table 1:** Copper ions’ effects on selected metal-binding proteins implicated in Alzheimer’s disease.

Protein.	Effect	Animal and Cell Model
Amyloid-β	Cu plays a role in modulating the aggregation of amyloid-β and decreases toxicity; nevertheless, the presence of copper insoluble amyloid-β accelerate apoptotic cell death. Sub-stoichiometric levels of copper(II are rendered Aβ aggregation and cause more neurotoxicity.	A synthetic peptide (Aβ2535), HEK293 cell,PC-12,and primary hippocampal cells [149,150,151,152,153,154].
Tau	Plays in modulating phosphorylation. Plays in modulating Aβ aggregation.	Triple-transgenic mice model of AD (3xTg-AD), SHSY5Y human neuroblastoma cells, and Alzheimer’s disease transgenic mouse model [155,156]. A peptide from tau possesses a repeat microtubule-binding domain [157].
Amyloid precursor protein	Increase expression levels and distribution of APP and amyloid-β, respectively. Copper has promoted traffic and redistribution of APP. Increases Cu^2+^ mediated oxidative stress as well as APP ectodomain neuronal cell death.	APP/PS1transgenic mice, N2a cells, primary cortical neurons, MDCK-APP-cherry cells, polarized epithelial cells, SH-SY5Y cells [112,115,156,158]. Recombination of amyloid precursor protein (APP), APP mutant cells, and primary neuronal cell lines [159].
